# A combined FSTRA-shotgun proteomics approach to identify molecular changes in zebrafish upon chemical exposure

**DOI:** 10.1038/s41598-019-43089-7

**Published:** 2019-04-29

**Authors:** Steve U. Ayobahan, Elke Eilebrecht, Matthias Kotthoff, Lisa Baumann, Sebastian Eilebrecht, Matthias Teigeler, Henner Hollert, Stefan Kalkhof, Christoph Schäfers

**Affiliations:** 1 Fraunhofer Institute for Molecular Biology and Applied Ecology IME, Schmallenberg, Germany; 2 Institute of Environmental Research (Biology V), RWTH Aachen, Aachen, Germany; 3 Aquatic Ecology & Toxicology, University of Heidelberg, Heidelberg, Germany; 4 Department 2, Hamm-Lippstadt University of Applied Sciences, Hamm, Germany; 5 Institute for Bioanalysis, University of Applied Sciences Coburg, Coburg, Germany

**Keywords:** Bioinformatics, Proteomics

## Abstract

The fish short-term reproduction assay (FSTRA) is a common *in vivo* screening assay for assessing endocrine effects of chemicals on reproduction in fish. However, the current reliance on measures such as egg number, plasma vitellogenin concentration and morphological changes to determine endocrine effects can lead to false labelling of chemicals with non-endocrine modes- of-action. Here, we integrated quantitative liver and gonad shotgun proteomics into the FSTRA in order to investigate the causal link between an endocrine mode-of-action and adverse effects assigned to the endocrine axis. Therefore, we analyzed the molecular effects of fadrozole-induced aromatase inhibition in zebrafish (*Danio rerio)*. We observed a concentration-dependent decrease in fecundity, a reduction in plasma vitellogenin concentrations and a mild oocyte atresia with oocyte membrane folding in females. Consistent with these apical measures, proteomics revealed a significant dysregulation of proteins involved in steroid hormone secretion and estrogen stimulus in the female liver. In the ovary, the deregulation of estrogen synthesis and binding of sperm to zona pellucida were among the most significantly perturbed pathways. A significant deregulation of proteins targeting the transcriptional activity of estrogen receptor (*esr1*) was observed in male liver and testis. Our results support that organ- and sex-specific quantitative proteomics represent a promising tool for identifying early gene expression changes preceding chemical-induced adverse outcomes. These data can help to establish consistency in chemical classification and labelling.

## Introduction

Substances that interfere with the endocrine system, and exert an adverse outcome on organism development and reproductive capability, are generally referred to as endocrine disrupting chemicals (EDCs). From a regulatory perspective, EDC definitions mainly focus on chemicals that trigger developmental and reproductive dysfunction, through interference with natural hormone production responsible for normal physiological processes^1^. In fact, many chemicals have been identified as EDCs with regard to their potential to interact with the endocrine system^2^. Endocrine disruptor screening programs (e.g. EDSP) have developed a battery of mechanism-specific *in vitro* and *in vivo* assays for screening and testing of chemicals in order to identify potential endocrine disruptors (EDs) and characterize their endocrine activity (specifically estrogen, androgen, thyroid, and steroid; EATS). In accordance with the guidance document for the identification of endocrine disruptors, the need to develop a biologically plausible mode-of-action (MoA) link between observed adverse effects and endocrine activity has been outlined. Such a method should take into account plausible interference between non-EATS endocrine activity and the observed adversity.

Current testing strategies have evolved significantly over the years. Existing standard methods need to be readjusted in order to cope with the continuously increasing testing needs. The recent scientific debate on the assessment of endocrine disrupting properties of chemicals has challenged the present testing practice. Additional weight of evidence is required to provide the causal links between the chemicals’ MoAs and its potential adverse effects^1,3^. The potential consequences of improper labelling of non-endocrine disruptors as EDCs and the resulting strict regulations on EDC hazard, have given rise to the need for a robust and integrative analytical method, which allows consistent identification and description of any MoA, ideally within one testing approach. The current reliance on endpoints such as egg number, plasma vitellogenin (VTG) content and morphological changes to determine effects of EDCs has generated a number of questions on how to handle chemicals interfering with the endocrine system as a secondary effect, operating via different MoAs.

Zebrafish (*Danio rerio*) has been shown to be a valuable model organism, commonly used in ecotoxicological research to study the effects of chemicals on aquatic ecosystems, biological functions and development^4–8^. It is one of the recommended species in a wide range of ecotoxicological OECD test guidelines for fish, including EDC testing (e.g. OECD TG 229; OECD TG 234). With consistency in endpoint assessment, zebrafish has been actively used in toxicity investigation that ranges from effects on development^9^, mutations and genotoxic effects^10^, behavioral alterations^11^ to reproduction^12–14^. However, the obtained information on the functionality and the disruption of the endocrine system is limited to EDCs affecting plasma VTG content, fertility and fecundity, and gonad health. The current FSTRA strategies focus on the assessment of chemicals regulating estrogen, androgen and steroidogenesis^15,16^. However, several non-EDCs could trigger processes that regulate the current indicators of endocrine activity. Considering the capability to quantify several hundred proteins simultaneously, shotgun proteomics shows appreciable promise as a molecular diagnostics tool to identify specific alterations and sex-specific physiological responses between exposures and controls, allowing a better understanding of a chemicals’ MoA. Investigating differentially expressed proteins by proteomics can provide in-depth understanding of cellular or tissue expression profiles and networks that could not be achieved by evaluating the expression of individual proteins.

To this end, a FSTRA according to OECD TG 229^15^ was performed with fadrozole, a known aromatase inhibitor, in combination with the identification of organ-and sex-specific molecular responses in zebrafish. Fadrozole is an anti-breast cancer drug^17^, which acts as a non-steroidal aromatase inhibitor that specifically inhibits estrogen synthesis^18^ and triggers adverse reproductive effects in fish^19–23^. Hence, fadrozole has been shown to be highly specific and shows an established endocrine disrupting MoA^18,24^. Its endocrine disrupting effects on fish are reasonably well defined^18–21,25–30^. Therefore, it provides a proof-of-principle model substance to evaluate and validate a shotgun proteomics approach as suitable tool for the integration of molecular methods into standard test guidelines.

This study further aimed at the identification and confirmation of alterations in molecular toxicity pathways that are specific to chemical-induced apical responses in zebrafish. It identifies sex-specific proteome responses to a primary impairment of estrogen synthesis as specific molecular perturbations resulting in adverse reproductive effects. The obtained molecular signatures could serve as initiating information for the development of a discrimination tool in endocrine disruptor testing.

## Results

### Effects of fadrozole on FSTRA endpoints

**Figure 1:**
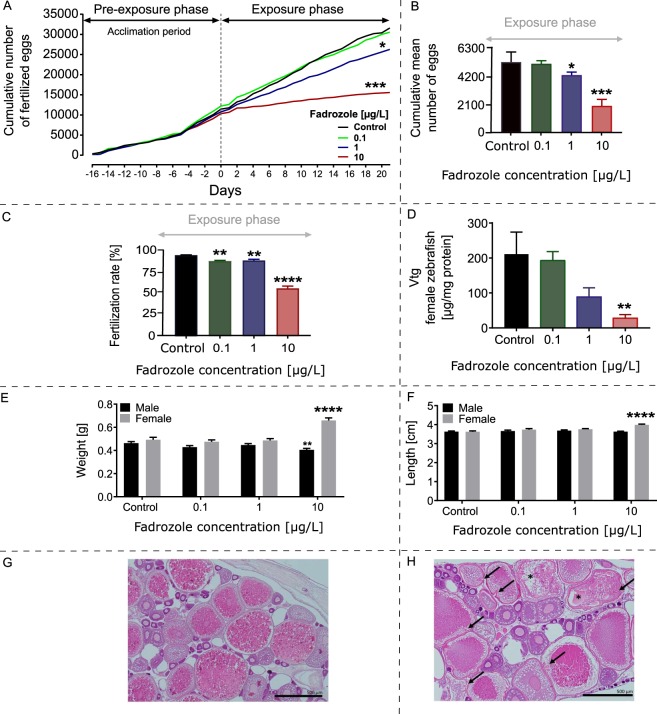
Physiological and histopathological effects of 21 days fadrozole exposure. (**A**) Cumulative fecundity of zebrafish during the 16 days acclimation period prior to treatment and within the subsequent 21 days exposure period. A statistically significant reduction in mean egg numbers was observed upon exposure to 1.0 µg/L and 10.0 µg/L fadrozole treatment as compared to the control group in the exposure phase. (**B**) The cumulative mean number of fertilized eggs within the 21 days exposure period was observed to be significantly reduced in the 1 µg/L and 10 µg/L fadrozole conditions. (**C**) Fertilization rate of zebrafish after the exposure to fadrozole for 21 days was observed to decrease significantly in a concentration-dependent manner. (**D**) Plasma VTG content was significantly reduced at 10 µg/L fadrozole treatment compared to control fish. Statistically significant differences were calculated by ANOVA, followed by Dunnetts post-hoc t-test with significance levels set at *p < 0.05, **p < 0.01, ***p < 0.001, ****p < 0.0001); n = 2 fish/replicate, 4 replicates/treatments. (**E**) Weight of male and female zebrafish exposed to fadrozole recorded a significant increase in the weight of female zebrafish and a decrease in weight for male zebrafish. (**F**) Length of male and female zebrafish exposed to fadrozole recorded a significant increase in the length of female; a no significant change was observed for male zebrafish. (**G**) Section of control ovary showing no pathological alterations. (**H**) Representative histopathological observation of female ovary at 10 µg/L fadrozole treatment showing mild oocyte atresia and membrane folding (Arrows: membrane folding; Asterisks: atretic oocytes).

The 21 days exposure of zebrafish to fadrozole resulted in no mortality neither in control nor in treatment samples. The cumulative mean number of eggs per female during the 16 days acclimation period directly preceding exposure did not differ in a statistically significant manner when comparing the fish from different tanks subsequently used for the different exposures (Fig. 1A). In the 21 days exposure phase, the number of eggs decreased in a concentration-dependent manner compared to the non-treatment controls (8.8%, 22.9% and 73.9% reduction at 0.1 µg/L, 1.0 µg/L and 10.0 µg/L respectively; Fig. 1A,B). The observed decrease was statistically significant at 1.0 and 10.0 µg/L fadrozole treatment (Dunnetts t-test, *p1.0 < 0.05, ***p10.0 < 0.001). The mean fertilization rate of the fish during the 21 days exposure to 0, 0.1, 1, and 10 µg/L fadrozole was 95.3 ± 0.6%, 88.3 ± 1.6%, 89.0 ± 2.5% and 54.7 ± 6.4%, respectively (Fig. 1C; Table S1; Dunnetts t-test, **p0.1 < 0.01, **p1.0 < 0.01, ****p10.0 < 0.0001).

### Vitellogenin measurement

The mean measured concentration of female plasma VTG was 211 ± 62.8, 192.9 ± 25.4, 88.4 ± 26.4 and 27.59 ± 10.5 µg VTG/mg protein (Mean ± SEM) for 0.0, 0.1, 1.0 and 10.0 µg/L fadrozole treatment, respectively. A statistically significant decrease in plasma VTG was observed for the 10.0 µg/L treatment condition (Dunnett, **p < 0.01; Fig. 1D).

Male zebrafish showed a decrease in weight in the exposed groups when compared to the non-treated control. The mean weight was recorded as 0.46 ± 0.01 g, 0.43 ± 0.02 g, 0.43 ± 0.01 g and 0.40 ± 0.01 g (mean ± SEM) for 0.0, 0.1, 1.0 and 10.0 µg/L fadrozole treatment. A statistically significant decrease in male weight was observed at 10 µg/L fadrozole treatment only (Dunnetts t-test, **p < 0.01). The mean distribution of male length after fadrozole exposure was recorded as 36.3 ± 0.4 mm, 36.6 ± 0.8 mm, 36.3 ± 0.4 mm and 36.3 ± 0.3 mm (mean ± SEM) for 0.0, 0.1, 1.0 and 10.0 µg/L fadrozole treatment, respectively.

The mean weight of female zebrafish observed after of 21 days exposure was 0.49 ± 0.02 g, 0.48 ± 0.02 g, 0.49 ± 0.02 g and 0.67 ± 0.02 g (mean ± SEM) upon 0.0, 0.1, 1.0 and 10.0 µg/L fadrozole treatment, respectively. Females treated with 10.0 µg/L fadrozole showed a statistically significant increase in weight (Dunnetts t-test, ****p < 0.0001; Fig. 1E). A similar trend was observed for the mean length of female fish at test end with 36.3 ± 0.4 mm, 37.4 ± 0.8 mm, 37.5 ± 0.5 mm, and 40.0 ± 0.5 mm upon 0.0, 0.1, 1.0 and 10.0 µg/L treatment, respectively (mean ± SEM). Also in this case the highest fadrozole concentration yielded a statistically significant increase in length (Dunnetts t-test, ****p < 0.0001; Fig. 1F).

### Histopathology

Based on the maturity index calculation concept introduced by Baumann *et al*. (2013)^31^ the average maturity index of females showed a slight, non-significant concentration-dependent increase from a mean value of 4.3 in non-treated controls to mean value of 4.6 upon 1.0 µg/L and 10.0 µg/L fadrozole treatment, respectively (data not shown). Histopathologically, mild oocyte atresia combined with oocyte membrane folding were observed in females upon 10.0 µg/L fadrozole treatment, but not in the other exposure groups (Fig. 1G showing a control ovary tissue, Fig. 1H showing a 10.0 µg/L fadrozole treated ovary tissue). In the male testis, maturity index showed a slight, non-significant concentration dependent decrease from a mean value of 3.9 in non-treated controls, 0.1 µg/L, and 1.0 µg/L to 3.5 upon 10.0 µg/L fadrozole treatment (data not shown).

### Organ and sex specific proteomic analyses

**Figure 2:**
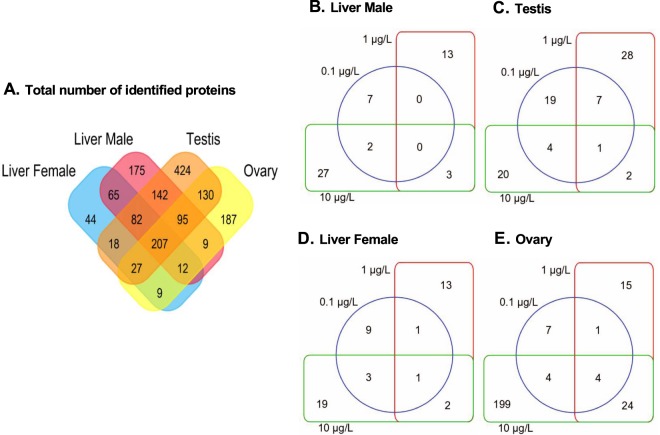
Venn diagram of identified proteins. (**A**) Proteomic analysis revealed that the total number of quantified proteins in the different tissues of zebrafish following 21 days of fadrozole exposure to groups of spawning zebrafish is 787 (male liver), 719 (testis), 464 (female liver) and 676 (ovary). (**B**–**E**) Venn diagrams showing the overlap between proteins being significantly regulated at different fadrozole concentrations in male liver (**B**), in testis (**C**), in the female liver (**D**) and in ovary. (**E**) Statistically significant differences were set at mean fold change >2, FDR of 5% and q ≤ 0.05.

To analyze if the observed concentration-dependent decrease in fecundity and VTG content was the consequence of a primary impairment in estrogen synthesis triggered by fadrozole, an organ- and sex-specific proteome analysis was performed using a bottom-up proteomic approach. Across all test conditions, 787, 1130, 464 and 676 proteins were quantified in zebrafish male liver, testis, female liver and ovary, respectively (Fig. 2A).

In male liver, a total of 9, 16 and 32 proteins was statistically significantly dysregulated when compared to the non-treated controls (q-value < 0.05) upon exposure to 0.1, 1.0 and 10.0 µg/L fadrozole, respectively (Fig. 2B). In male testis, 31, 38 and 27 proteins were statistically significantly differentially expressed compared to the non-treated control group (q-value < 0.05; Fig. 2C). A similar trend in protein regulation was also observed in female liver, where 14, 17 and 25 proteins were statistically significantly dysregulated upon treatment with 0.1 µg/L, 1.0 µg/L, and 10.0 µg/L fadrozole compared to the control, respectively (q-value < 0.05; Fig. 2D). A concentration-dependent increase in the number of differentially expressed proteins was also detected in female ovary, where 16, 44 and 231 proteins were statistically significantly dysregulated when compared to the control upon treatment with 0.1 µg/L, 1.0 µg/L, and 10.0 µg/L fadrozole, respectively (q-value < 0.05; Fig. 2E).

### Specific dysregulated molecular responses by fadrozole exposure across tissues

To profile fadrozole-perturbed signaling pathways in different tissues, a pathway enrichment analysis was performed based on the tissue-specific protein expression changes. The presented data depict the comparison between controls and the treatment with 10 µg/L fadrozole.

**Figure 3:**
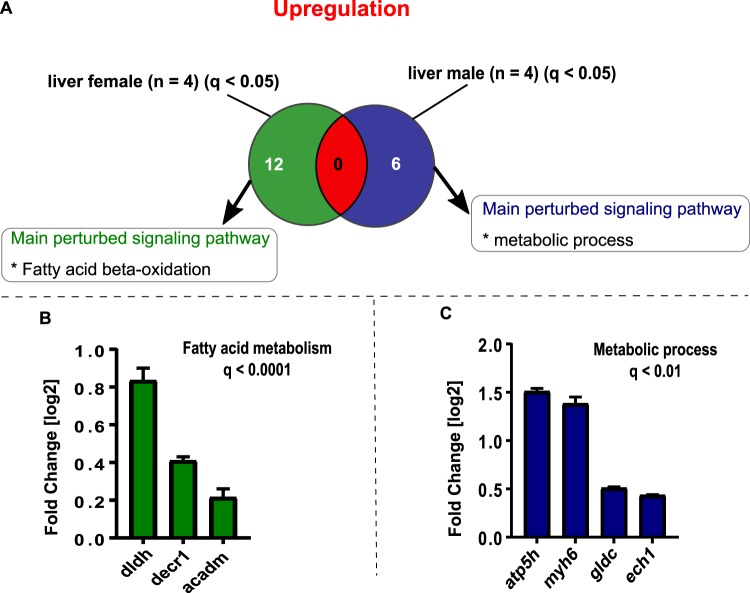
A comparative venn diagram of significantly upregulated proteins (log2FC corresponding to q-value < 0.05) investigated by TMT quantitative proteomic approach for both female and male zebrafish liver following 10 µg/L fadrozole exposure for 21 days (see Supplementary excel table). Significantly upregulated female and male liver proteins are depicted in green and blue, respectively. The number of commonly regulated proteins is shown in red. (**A**) Upregulated proteins in the female liver were involved in fatty acid metabolism. (**B**) Metabolic process was the main upregulated signaling pathway in the male zebrafish liver. Statistically significant enrichment clustering of differentially expressed proteins were set at FDR of 5% and q ≤ 0.05.

**Figure 4:**
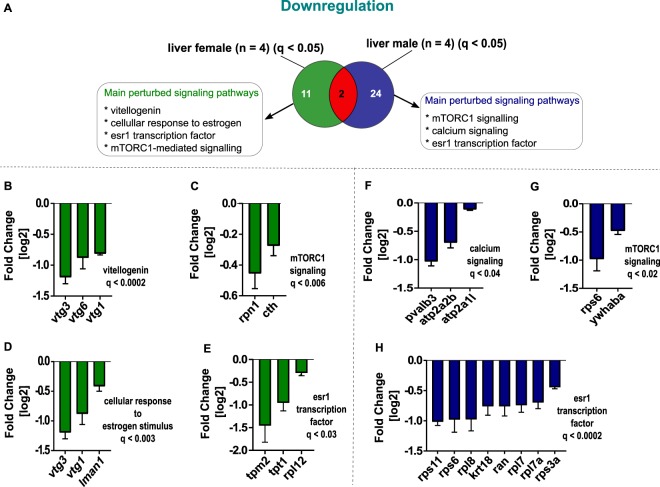
A comparative venn diagram of significantly downregulated proteins (log2FC corresponding to qvalue < 0.05) investigated by TMT quantitative proteomic approach for both the female and male zebrafish liver following 10 µg/L fadrozole exposure for 21 days. Significantly downregulated female and male liver proteins are depicted in green and blue, respectively. The number of commonly regulated proteins is shown in red. (**A**) Molecular functions downregulated in the female liver were vitellogenin (**B**), mTORC1 signaling (**C**), cellular response to estrogen stimulus (**D**) and esr1 transcription factor (**E**). Calcium signaling (**F**), mTORC1 signaling (**G**) and the esr1 transcription factor signaling (**H**) were the main downregulated pathways in the male zebrafish liver. Statistically significant pathway enrichment-based clustering of differentially expressed proteins were set at FDR of 5% and q ≤ 0.05.

In female and male zebrafish liver, different pathways were affected (Figs 3A and 4A). In females, the exposure to 10 µg/L fadrozole leads to an upregulation of fatty acid metabolism (Fig. 3B), while in males the main upregulated pathway was metabolic process (Fig. 3C). In female liver, vitellogenin (Fig. 4B), the mechanistic target of rapamycin complex 1 (mTORC1) signaling (Fig. 4C), estrogen stimulus (Fig. 4D) and *esr1* transcription factor (Fig. 4E) were statistically significantly downregulated. The main downregulated signaling pathways in male liver are involved in calcium signaling (Fig. 4F), mTORC1-mediated signaling (Fig. 4G) and *esr1* transcription factor inhibition (Fig. 4H).

**Figure 5:**
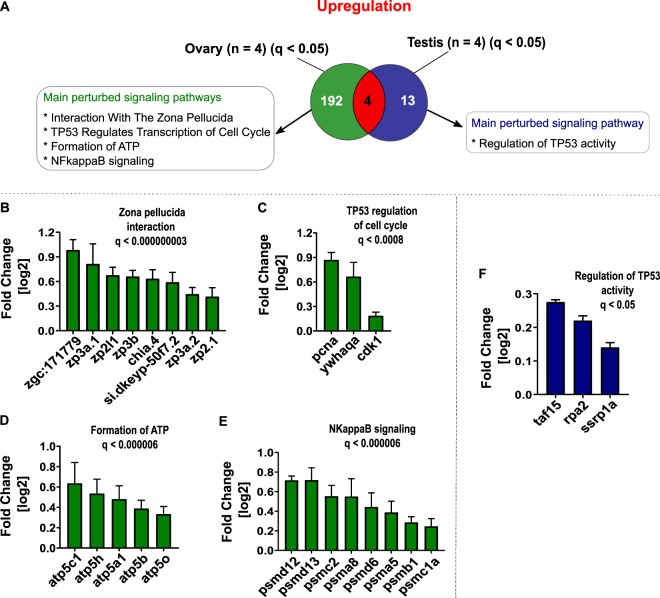
A comparative venn diagram of significantly upregulated proteins (log2FC corresponding to qvalue < 0.05) investigated by TMT quantitative proteomic approach for both the ovary and testis following 10 µg/L fadrozole exposure for 21 days. Significantly upregulated ovary and testis proteins are depicted in green and blue, respectively. The number of commonly regulated proteins is shown in red. (**A**) Significantly, upregulated proteins in the ovary were involved in zona pellucida interaction (**B**), TP53 regulation of cell cycle (**C**), formation of ATP synthesis (**D**) and NkappaB signaling (**D**). Regulation of TP53 activity was the main upregulated pathway in testis. (**F**) Statistically significant enrichment-based analysis of differentially expressed proteins were set at FDR of 5% and q ≤ 0.05.

**Figure 6:**
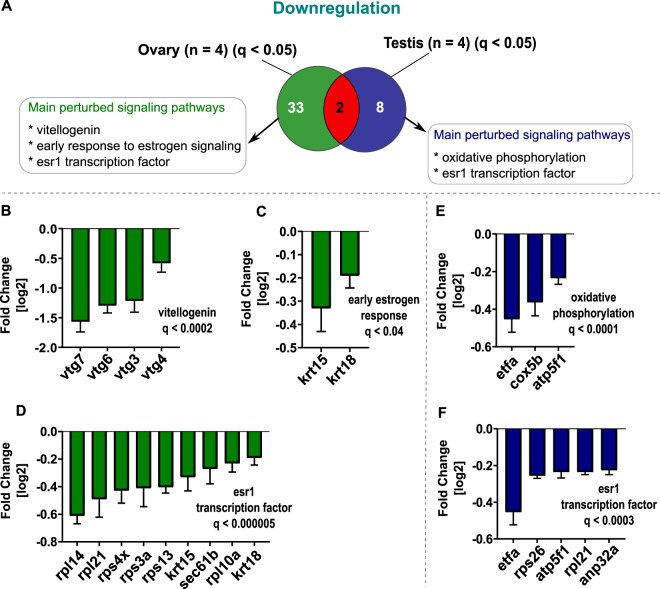
A comparative venn diagram of significantly downregulated proteins (log2FC corresponding to qvalue < 0.05) investigated by TMT quantitative proteomic approach for both ovary and testis following 10 µg/L fadrozole exposure for 21 days. Significantly downregulated ovary and testis proteins are depicted in green and blue, respectively. The number of commonly regulated proteins is shown in red. (**A**) Downregulated proteins in the ovary were vitellogenin (**B**), cellular response to estrogen stimulus (**C**) and the esr1 transcription factor signaling. (**D**) Oxidative phosphorylation (**E**) and the esr1 transcription factor signaling were the main downregulated pathways testis (**F**). Statistically significant pathway enrichment-based clustering of differentially expressed proteins were set at FDR of 5% and q ≤ 0.05.

We next compared up- and downregulated pathways in gonads (Figs 5A and 6A). Upregulated molecular pathways in ovary include the zona pellucida interaction (Fig. 5B), regulation of p53 tumor suppressor protein (Fig. 5C), formation of ATP synthesis (Fig. 5D) and NfkappaB signaling (Fig. 5E). The regulation of p53 tumor suppressor protein was also upregulated in testis (Fig. 5F). Significantly downregulated pathways in ovary were vitellogenin (Fig. 6B), early estrogen response (Fig. 6C) and *esr1* transcription factor signaling (Fig. 6D). In testis, molecular function involved in oxidative phosphorylation (Fig. 6E) and *esr1* transcription factor signaling (Fig. 6F) were significantly downregulated.

## Discussion

This study evaluates the efficiency of shotgun proteomics for the identification of organ- and sex-specific molecular responses in zebrafish following aromatase inhibition. Furthermore, this investigation sheds light on the implication of aromatase inhibition in reproductive dysfunctions. The main idea was to test the applicability of an integration of proteomics in OECD standard test approaches. The resulting combined approach could provide the causal link between the chemicals’ MoA and the resulting adverse effect. As a proof-of-principle, we treated both male and female zebrafish for 21 days with fadrozole, a substance with known endocrine MoA^20,30^. The concentrations of fadrozole (0.1, 1.0 and 10.0 µg/L) used in the study were unlikely to trigger systemic toxicity and lethality among the exposed fish. Indeed, no lethality was observed during the 21 days treatment, indicating the exposure concentrations used to be below the threshold for systemic toxicity. We observed a concentration-dependent reduction in the number of eggs as well as a decrease in plasma VTG content in females (Fig. 1A,D), which is in accordance with the effects reported for fathead minnow (*Pimephales promelas*) after exposure to fadrozole^20^. A reduction in fecundity has been linked to decreased plasma VTG concentrations. This is an indication for a decreased estrogen synthesis, which is responsible for reproductive viability in fish^18,20,32^. Our findings further agree with previous fadrozole exposure studies resulting in the reduction of VTG content and egg numbers^1^. In addition to these responses, female zebrafish exposed to 10.0 µg/L fadrozole showed a slight increase in weight as compared to the non-treated controls, which may be explained by the fact that the females were more mature and more eggs were retained in their body due to their impaired reproductive ability (Fig. 1A). The observed pathological alterations also confirm this hypothesis that eggs were old and started degenerating (Fig. 1H). Accompanying these responses, fadrozole exposure was observed to have a slight stimulating effect on female gonad maturity and a slight inhibiting effect on male gonad maturity. However, no pathological alterations of testicular morphology could be detected in any of the treatment groups, whereas a clear treatment-related effect was detected in female ovary. The observed oocyte membrane folding in females (Fig. 1H) can be regarded as presumably degenerative (atretic) process characterized by abrupt, usually multiple, invaginations of the chorion. This is an effect, which has been reported for adult female zebrafish after exposure to the anti-estrogen tamoxifen^33,34^ and thus is likely to be mediated by EDCs with androgenic, anti-estrogenic or aromatase-inhibiting MoAs. All fish displayed normal liver structure with average glycogen levels and lipid amounts showing that the treatment did not induce hepatotoxic side effects.

This study provides additional molecular data on the dysregulated biological processes upon aromatase inhibition, as complementary information to the observed apical effect. Compared to the experimental control, proteomics identified the highest number of significantly dysregulated proteins (q < 0.05) following 10 µg/L fadrozole exposure in the ovary (Fig. 2E), which affirms the observed reproductive impairment. Thus, proteomics clearly revealed a distinguishable sex- and organ-dependent molecular response of zebrafish to fadrozole exposure, indicating the ovary to be the most affected organ, which is in agreement with the MoA.

To reveal the perturbed molecular patterns in the ovary underlining impaired reproduction, which resulted in the reduced egg numbers (Fig. 1A), we performed a knowledge-based functional annotation analysis with significantly dysregulated proteins (q < 0.05). In ovary, functional annotation clustering of dysregulated proteins at 10.0 µg/L fadrozole exposure revealed effects on the downstream transcription rate of estrogen receptor (*esr1)*, as involved proteins such as ribosomal proteins *(rpl14*, *rpl21*, *rps4x*, *rps3a*, *rps13*, *rpl10a)*, protein transport protein Sec61 subunit beta *(sec61b) and* keratin proteins *(krt15*, *krt18)* were observed to be downregulated (Fig. 6D). The deregulation of keratin proteins (*k15* and *k18)* (Fig. 6C) further indicates an early impairment in estrogen signaling.

This molecular response pattern was reinforced by the significant downregulation of proteins targeting the *esr1* transcription start site in the female liver (Fig. 4E), including tropomyosin 2 (*tpm2*), translationally controlled tumor protein (*tpt1*) and ribosomal protein L22 (*rpl22*). Furthermore, the *esr1* downstream transcription was impaired also in the male liver via the deregulation of proteins such as ribosomal proteins (*rps11*, *rps6*, *rpl8*, *rpl7*, *rpl7a*, *rps3a)*, keratin 18 *(krt18)*, and GTP-binding nuclear protein (*ran*) (Fig. 4H). In testis, a transcriptional regulation of *esr1* downstream activity was observed as well, as a deregulation of ATPase inhibitor A (*atp5f1)*, electron transfer flavoprotein subunit alpha (*etfa)*, as well as ribosomal proteins (rps26, *rpl21)* (Fig. 6F) was quantified.

Accompanying these molecular perturbations, enrichment analysis also revealed a significant downregulation of vitellogenin such as *vtg3*, *vtg4*, *vtg6*, *vtg7* in the ovary (Fig. 6B). Vitellogenin is a known biomarker for EDC testing and its expression was confirmed by ELISA analysis of plasma VTG concentrations in exposed female zebrafish (Fig. 1D). Similarly, the downregulation of vitellogenin (*vtg1*, *vtg3*, *vtg6*) (Fig. 4B) was also detected in the female liver. In addition, the observed downregulation of lectin, mannose binding, 1 *(lman1*, in combination with *vtg1* and *vtg3*) (Fig. 4D) in the female liver, a protein which is required for female sexual development^35^, further suggests an impairment in estrogen synthesis. The maintenance of VTG concentration in fish has been linked to an impairment in estrogen synthesis and a corresponding estrogen receptor activation, resulting in a decrease in VTG content^20^. The deregulation of vitellogenin observed by our proteome analysis also validates the reduced plasma VTG concentrations measured via ELISA analysis.

To achieve fertilization, sperm cells need to first interact with the eggs’ zona pellucida^36^. The dysregulation of proteins associated with the binding of sperms to the zona pellucida, which is a critical step for fertilization to occur, further highlighted the specificity of fadrozole exposure on reproductive impairment in zebrafish. The zona pellucida is an extracellular matrix composed of glycoproteins such as zp1, zp2, and zp3. *Zp3* proteins have been reported to function as the primary sperm receptors that induce the acrosome reaction^37^. In our study, the proteins involved in zona pellucida interaction (*zgc:171779*, *zp3a*.*1*, *zp2l1*, *zp3b*, *chia*.*4*, *si:dkeyp-50f7*.*2*, *zp3a*.*2*, *zp2*.*1*) were observed to be significantly upregulated in the zebrafish ovary subsequent to 10 µg/L fadrozole exposure (Fig. 5B), which implies a dysregulation of the fertilization processes. In fact, zona pellucida proteins, such as *zp3a*.*1*, *si:dkeyp-50f7*.*2*, *zp3b* (see supplementary excel table on ovary), were also upregulated upon 1 μg/L fadrozole treatment, although this perturbation was subtle, but concomitant with the observed decreased in egg numbers (Fig. 1A), as also shown in part by previous studies^18,20,38^. Based on these findings, the main adverse outcome of fadrozole exposure in adult zebrafish can be deduced to be the dysregulation of reproductive functions (Fig. 1A–D,H). Notably, the observed perturbation of proteins involved in zona pellucida interaction in the ovary (Fig. 5B), thus reflects a clear response to the impairment in estrogen-dependent VTG synthesis (Fig. 1D)^39–41^.

Upregulated pathways were rather associated with more general responses to adverse stimuli than to estrogenic signaling. For example, the tumor suppressor p53 is a transcription factor that controls several biological processes such as cell cycle, growth, apoptosis and genome stability^42,43^ and has been previously reported to enhance metabolic pathways that are anti-estrogenic such as fatty acid oxidation, thereby inhibiting tumorigenesis^44^.

Functional annotation analysis further detected the upregulation of proteins involved in nuclear factor kappa-light-chain-enhancer of activated B cells (NF-ҝB) signaling (Fig. 5E), which is an indication of inflammatory processes. The activation of NF-ҝB signaling pathway has been linked to inflammation and reproductive disorder^45^, which is in line with the observed apical effect of reduced egg numbers and histopathological alterations in the ovary, as observed in this study (Fig. 1A,H).

Thus, across all tissues, our study revealed dysregulated proteins associated with the downregulation in estrogen synthesis. These proteins, for example, could serve as biomarkers for gene expression studies of impaired reproductive function. These observations further provide hints to the MoA of fadrozole and support the suitability of proteomics as a tool for the provision of causal links between MoA and adverse effect. By developing an integrative approach that takes into account proteomic analysis together with standard examinations, we might be able to detect new biomarkers and address the robustness and deepness in EDC assessment. Interestingly, proteomics did not reveal any response to aromatase inhibition on the aromatase concentration itself. This could be contributed to the physiology of the test species zebrafish, which does not respond by compensatory mechanisms, but rather by a prompt physiological response, resulting for example in a skewed sex ratio, if disturbed during sexual differentiation, rather than an upregulation of aromatase^30^.

Taken together, this study demonstrates that a proteomics-based analysis of organ-and sex-specific responses is capable of detecting molecular dysregulation following fadrozole exposure. It also demonstrates the underlying alteration in biological functions, in relation to physiological responses attributed to the impairment in estrogen synthesis and reproduction. These results are in agreement with previous observations and established adverse effects for aromatase inhibition^18,20^. Our findings at the proteome level corroborate the observed apical endpoints such as the concentration-dependent reduction in fecundity, a reduction in plasma VTG concentrations, oocyte atresia and the membrane folding preventing the oocyte from developing to maturity. This study points out that a quantitative proteomic approach can serve as a sensible tool for the identification of molecular key events and biomarkers specific for a chemical’s MoA. These data provide an effective and efficient analytical basis for the development of a molecular screening. Thus, the use of quantitative proteomics in assessing (eco)toxicity in the future will critically improve chemical classification. Our findings underline the need for an integration of omics data into new testing strategies to identify chemical-induced, MoA-specific toxicity pathways, thereby establishing causal links between substances’ MoA and the resulting adverse effects.

## Materials and Methods

### Test species

Wild-type zebrafish (*Danio rerio*) originally obtained from West Aquarium GmbH (Bad Lauterberg, Germany) and continuously bred for several generations at the Fraunhofer IME laboratories, were used for testing. Fish of the broodstock were maintained under flow-through conditions in 150 L tanks at 25 ± 2 °C on a 12:12 h light/dark cycle in a temperature-controlled room. They were fed daily with TetraMin® (Tetra Werke, Melle, Germany) main feed *ad libitum* and nauplii of *Artemia salina*.

### Test substance

Fadrozole (IUPAC-Name 4-(5,6,7,8-Tetrahydroimidazo[1,5-a]pyridine-5-yl)-benzonitrile), was purchased from Adooq Bioscience LLC. (Irvine, Canada). The substance was directly dissolved in water for the preparation of the application solutions.

### Analytical determination of fadrozole concentrations

Water samples of 10 mL were taken from each tank from the mid water body, at test start, and once a week thereafter. Water samples were stabilized by addition of acetonitrile (1:1; v + v) containing 0.2% formic acid. Analysis of fadrozole concentrations was performed by high performance liquid chromatography tandem mass spectrometry (LC–MS/MS) with negative ionization. Data were collected on a Waters 2695 separation module coupled to a Quattro-Micro tandem mass spectrometer (Waters, Germany). Detailed information is provided in the supporting information section.

### Determination of FSTRA (OECD TG 229) endpoints

The FSTRA was conducted in accordance to the OECD TG 229^15^. Minor modifications of the protocol were necessary to include proteome analysis at test end. The test was performed with three test concentrations (0.1 µg/L, 1.0 µg/L, and 10.0 µg/L) and a dilution water control (See Table S1, Supporting Information), with four replicate vessels per concentration were run in parallel. Groups of ten zebrafish (5 males, 5 females) per vessel were exposed for 21 days. Survival, behavior and fecundity were assessed before and during the test by daily monitoring and quantitative egg counts. At the end of the 21 days of fadrozole exposure, body weight and length were recorded (n = 10 per test vessel; 16 vessels in total). Blood samples were collected from two male and female fish from each of the four replicates per concentration and controls by cardiac puncture for the measurement of VTG. Plasma VTG concentrations were determined using an enzyme-linked Immunosorbent assay (ELISA) for zebrafish VTG (TECOmedical, Switzerland) in accordance with the manufacturers’ instructions. Whole bodies of the same fish were fixed in modified Davidsons’s fixative (7.4% formaldehyde, 10% glycerol, 30% ethanol, and 10% acetic acid) overnight, before being transferred to 10% neutral buffered formalin. Samples were automatically dehydrated, and embedded in paraffin with trunks ventrally orientated to the cutting surface. 4–5 µm thick sections of gonads and livers were produced and stained with hematoxylin-eosin for subsequent histopathological investigations according to the OECD histopathology guidance document^46^. The sections were screened for the following histopathological lesions of the liver: hepatocyte necrosis, hepatocellular vacuolation, cystic degeneration, hyalinized hepatocytes and inflammatory cells. These criteria were based on the assessment of hepatic lesions in rodents according to Thoolen *et al*. (2010), with adaptations for fish as proposed by Schmidt-Posthaus *et al*.^47,48^. The livers and gonads of the remaining three males and females per replicate were isolated, and the individual organs of the three fish of each sex were pooled. The tissues were stored at −80 °C until protein extraction.

The experimental protocols were approved by the State Agency for Nature, Environment and Consumer Protection (LANUV), North Rhine Westphalia, Germany (reference number 84-02.04.2016.A097). The experimental work was conducted in accordance to these protocols and to the German Animal Welfare regulations (TierSchG).

### Protein extraction and analysis

Tissues were homogenized using a motor-driven plastic pestle in 6 M urea, 2 M thiourea, 4% CHAPS (3-[(3-cholamidopropyl)dimethylammonio]-1-propanesulfonate hydrate) in 50 mM TEAB (Triethylammonium bicarbonate), at pH 8.2. Afterwards, 100 µg of protein per treatment was subjected to reduction through the addition of a reducing agent with 200 mM TCEP (tris-(2-carboxyethyl)phosphine, hydrochloride). Subsequently proteins were alkylated with 375 mM iodoacetamide followed by overnight tryptic digestion at 37 °C. The resulting peptides from the various replicates of fadrozole treatment were labeled using a TMT-6-plex (see Supplementary Table S2) as recommended by the manufacturer. Samples were injected on a separating column and the peptides were eluted using a linear gradient from 5–95% acetonitrile, 0.1% formic acid with a flow rate of 300 nL/min and monitored using Q Exactive mass spectrometer. Detailed information is provided in the supporting LC-MS/MS analysis section. The mass spectrometry proteomics data have been deposited to the ProteomeXchange Consortium via the PRIDE^49^ partner repository with the dataset identifier PXD011022.

### Bioinformatics of proteome data

LC-MS raw data were processed using proteome discoverer 2.2 software (Thermo Fisher Scientific, Germany). For peptide identification, MS data were matched to a zebrafish database obtained from Ensembl (Download version: April. 02. 2017) using the SEQUEST algorithm. During identification, carbamidomethylation (fixed modification) and methionine oxidation as well as N-terminal acetylation (both dynamic modifications) and up to 2 enzymatic mis-cleavages were considered (Fig. S1A, Supporting Information). Proteins were considered as identified if at least two unique peptides were matched. Normalization was performed to correct experimental bias using total peptide amount (Fig. S1B, Supporting Information). The ratio of normalized control to treated data were log2-transformed for downstream analysis. To control error in statistical testing with proteomic data, false discovery rate (FDR) correction was incorporated during data analysis along with the various biological replicates. Multiple t-tests with FDR of 5% were applied to determine statistical differences. Adjusted p-value (q < 0.05) was considered statistically significant^50^. Functional enrichment analysis of differentially expressed proteins was conducted with DAVID bioinformatics tools^51^ and Panther gene list analysis^52^. Transcription factor analysis was performed using Enrichr web-based tool for intuitive enrichment analysis^53^ in order to identify common biological interactions and transcriptional targets from significantly regulated proteins.

### Statistical analyses for FSTRA endpoints

The number of replicates was based on the number of vessels per concentration. Values for single fish in replicates for the endpoints length, weight, VTG content, and histopathology were first averaged. Mean measured values (±SEM) provided in this publication were calculated based on the values for the vessel replicates. All statistical analyses of the data were performed using GraphPad Prism and R. The D’Agostino-Pearson (K2) and Shapiro-Wilks (W) tests were employed to evaluate normality of the data. Forsythe test was conducted to determine homogeneity of variances. Two-way analysis of variance (ANOVA), followed by Dunnett’s multiple comparisons tests (D_mc_) were used to determine statistical differences between experimental data of control and fadrozole treatment groups. Statistically significant differences were set at the *p ≤ 0.05, **p ≤ 0.01, ***p ≤ 0.001 and ****p ≤ 0.0001 levels.

## Supplementary information

Supporting Manuscript

Liver Female

Liver Male

Ovary

Testis

Fadrozole_analysis

Table S1
