# Supplementary material for: A combined FSTRA-shotgun proteomics approach to identify molecular changes in zebrafish upon chemical exposure
Source: Sci Rep. 2019 Apr 29;9:6599. doi: 10.1038/s41598-019-43089-7 (PMC6488664; doi:10.1038/s41598-019-43089-7)
Supplement: Supplementary file 1 — Supporting Manuscript [file 41598_2019_43089_MOESM1_ESM.pdf]

Supporting Information

A combined FSTRA-shotgun proteomics approach to identify molecular changes in zebrafish upon chemical exposure

Steve Ayobahan[IЖ, φ*], Elke Eilebrecht[IЖ], Matthias Kotthoff[¥], Lisa Baumann[Ж], Sebastian Eilebrecht[IЖ], Matthias Teigeler[IЖ], Henner Hollert[φ], Stefan Kalkhof[ʣ], Christoph Schäfers[IЖ]

[IЖ]Fraunhofer Institute for Molecular Biology and Applied Ecology IME, Schmallenberg, Germany.

[φ]Institute of Environmental Research (Biology V), RWTH Aachen, Germany

[Ж]Aquatic Ecology & Toxicology, University of Heidelberg, Germany

[¥]Lippstadt University of Applied Sciences, Department 2, Marker Allee 76–78, 59063 Hamm, Germany

[ʣ] Institute for Bioanalysis, University of Applied Sciences Coburg, Germany

[*]**Authors for correspondence:** Steve Ayobahan, Email: steve.ayobahan@ime.fraunhofer.de, Elke Eilebrecht, Email: elke.eilebrecht@ime.fraunhofer.de, Tel. 02972 302-102, Fraunhofer Institute for Molecular Biology and Applied Ecology IME, Auf dem Aberg 1, 57392, Schmallenberg, Germany

13 pages, 3 figures, 2 tables

Summary

1) **General exposure conditions**
2) **Analytical determination of fadrozole concentrations**
3) **Result of chemical analysis of fadrozole concentrations**
   - **Table S1. Mean measured concentrations of fadrozole during the 21-days exposure (LOQ=0.05µg/L)**
4) **TMT-based quantitative proteomic analysis**
   - **Table S2. Quantitative TMT-6plex labelling mixing scheme for liver and gonad of zebrafish exposed to fadrozole for 21-days**
   - **Figure S1. Quality control of mass spectrometry data**
5) **Functional enrichment analysis of biological processes across different tissues**
   - **Figure S2. Enrichment analysis of regulated functional biological processes following 21days of fadrozole exposure**
   - **Figure S3. Comparative sex-specific enrichment analysis of regulated functional biological processes and activated pathways**

1) **General exposure conditions.** Purified tap water was used according to the OECD TG 229 [1]. The purification included filtration with activated charcoal. The water was aerated to the level of oxygen saturation. The water quality was monitored regularly in the testing facility. Water temperature in the test vessels was adjusted to 26 ± 2°C. Oxygen saturation of the test solutions was between 88.5 % and 92.0 %. The pH was between 7.7 and 7.8 in the test tanks. Adult zebrafish aged twelve months, were used in this study in accordance with the animal welfare standard practice at the Fraunhofer IME in Schmallenberg. Healthy fishes without any signs of disease were used for the test. Monitoring (health status, egg number) and acclimatization of selected tests animals prior

to fadrozole exposure were carried out for 2 weeks. The flow rate was fixed to a daily five-fold exchange, with a constant volume of 10 L in each tank. No physical signs or negative effects of fadrozole treatment were observed and all the zebrafish survived the three-week exposure period.

2) **Analytical determination of fadrozole concentrations.** Water samples of 10 mL volume were taken from each tank from the mid water body, at test start, and thereafter once a week. Water samples were stabilized by addition of acetonitrile (1:1; v + v) containing 0.2 % formic acid prior to storage. Fadrozole analysis was performed by high performance liquid chromatography tandem mass spectrometry (LC–MS/MS) with negative ionization. Data were collected on a Waters 2695 separations module coupled to a Quattro-Micro tandem mass spectrometer (Waters, Germany). Aliquots of 50 µL were injected into a Gemini C18 high-performance LC column (150 mm × 3 mm, 5 µm particle size; Phenomenex) at a flow rate of 0.5 mL/min and a column temperature of 30°C. Matrix-free procedural blanks were run each working day to exclude possible cross-contaminations during laboratory work. Separation was performed on a binary gradient of 20-mmol ammonium acetate solution in (A) methanol and (B) a 90:10 water:methanol mixture. The gradient started with 60 % A, increasing to 100 % A within 3 min, then returning to 60 % A and 40 % B after 6.1 min, and holding initial conditions for 3 min. A Calibration was performed in a concentration range of 0 µg/L to 250 µg/L fadrozole. The coefficient of correlation (r^2) of the calibration function was estimated ≥ 0.99. The substance-specific limit of quantitation (LOQ) was defined as 0.05 µg/L fadrozole.

3) **Result of chemical analysis of fadrozole concentrations.** No fadrozole traces were measured in the control water. Fadrozole concentrations were stable and did not vary by more than 20% from the target concentrations of 0.1, 1.0 and 10.0 µg/L. The measured mean concentrations were 0.099 ± 0.0004 (99.7 % recovery), 0.964 ± 0.028 (96.4 % recovery) and 9.974 ± 0.046 µg/L (99.7 %

recovery) over the test period (see Table S1). The coefficient of variation indicates low variability across treated vessels.

Table S1: Mean measured concentrations of fadrozole during the 21-days exposure (LOQ=0.05µg/L).

Treatment	Nominal-concentration [µg/L]	Measured mean fadrozole concentration [µg/L]	Recovery [%]
**Control**	<LOQ	<LOQ	-
**Concentration 1**	0.1	0.099 ± 0.0004	99.681 ± 0.365
**Concentration 2**	1	0.963 ± 0.0282	96.385 ± 2.820
**Concentration 3**	10	9.974 ± 0.0455	99.7400.455

Remark: The fadrozole concentrations were measured four times with seven days interval, in the four replicates per each treatment during the test duration.

4) TMT-based quantitative proteomic analysis

- **Protein Extraction**. Samples of liver and gonad tissues were pooled separately per treatment for male and female zebrafish, from each of the four biological replicates. All tissues were homogenized using motor-driven plastic pestles kontes (vwr) in a lysis buffer (6 M urea, 2 M thiourea, 2 g CHAPS detergent (3-((3-cholamidopropyl) dimethylammonio)-1-propanesulfonate) in 50mM TEAB (Triethylammonium bicarbonate), pH 8.2), with complete protease inhibitors (Merck Millipore). The tissue lysates were centrifuged at 13,000 g for 10 mins, the supernatants carefully transferred and the protein concentration measured with the NanoDrop 2000 (Thermo Scientific) using a Bradford protein assay (Bio-Rad, Germany). Protein extracts were concentrated and desalted by ultrafiltration devices (molecular weight cut-off 3 kDa) (Merck Millipore) at 18°C and centrifuged at 14,000 g for 10 mins against a buffer solution of 100mM TEAB.

- **Protein Digestion.** For in-solution digestion, 100 µg of protein per sample were transferred into a new Eppendorf tube with an addition of 5 µL of 10 % sodium dodecyl sulfate (SDS) (Thermo Scientific) for enhanced protein precipitation. The final volume was adjusted to 100 µL with 100 mM TEAB. Reduction of disulfide bonds was performed with 5 µL of 200 mM TCEP (Tris-(2-Carboxyethyl) phosphine, Hydrochloride; Thermo Scientific) for 1 h at 55°C. Subsequently proteins were alkylated with 375 mM iodoacetamide (IDD; Thermo Scientific) in the dark at room temperature for 30 minutes. For protein precipitation, 1 mL of pre-chilled (-20°C) methanol was added and the alkylated sample were incubated overnight at -20°C. The samples were quickly centrifuged at 8,000 g for 12 min at 4°C and the supernatant was decanted. Proteins were re-suspended with 100 µL of 100 mM TEAB. Enzymatic cleavage was conducted using 2.5 µg of trypsin per 100 µg of protein. Digestion was performed overnight at 37°C.
- **TMT Labelling.** Protein digestion into peptides using trypsin was immediately followed by TMT labelling. Immediately before use, the TMT labelling reagents were equilibrated to room temperature. Subsequently, 41 µL of anhydrous acetonitrile was added to each TMT labelling vial. The reagent was allowed to dissolve with occasional vortexing.100 µL of protein digest was added into the labelling vial (Table S2) and allowed to incubate for 1 hour at room temperature. Afterward, 8 µL of 5% hydroxylamine (Thermo Scientific) was added to each sample, followed by an incubation for 15 mins to stop the reaction. The labelled samples of four biological replicates per treatment were randomly combined at equal amounts in a new tube (Table S2). Thereafter, the labelled peptides were desalted using spin columns equipped with C18 reversed phase material by centrifugation at 1500 g for 1 min. Desalted peptides were eluted with 70 % acetonitrile followed by a gentle drying in a vacuum evaporator.

Table S2. Quantitative TMT-6plex labelling mixing scheme for liver and gonad of zebrafish exposed to fadrozole for 21-days.

	Combination			
Used TMT Labelling Reagent	126	127	128	129
Replicate 1	Control	0.1 µg/L	1 µg/L	10 µg/L
Replicate 2	10 µg/L	Control	0.1 µg/L	1 µg/L
Replicate 3	1 µg/L	10 µg/L	Control	0.1 µg/L
Replicate 4	0.1 µg/L	1 µg/L	10 µg/L	Control

***Remark**: A varying mixing scheme with channels 126, 127, 128 and 129 of TMT 6plex Isobaric Mass Tag, for enhanced multiplex relative quantitation was used to label the four replicates per treatment per tissue.*

- **LC-MS/MS Analysis.** The dry labelled TMT samples were re-suspended in 30 µL in 2 % acetonitrile and 0.1 % formic acid for LC-MS/MS analysis. The peptide samples were injected on a separating column (nanoAquity UPLC Column HSS T3, 75µm × 250mm, 1.8µm, Waters). Elution was performed over a 150 minute gradient at a flow rate of 300 nL/min using a linear gradient with mobile phase B ranging from 5 % to 95 % (mobile phase A: 0.1 % formic acid in water, mobile phase B: 0.1 % formic in acetonitrile). All samples were analyzed using a Q Exactive mass spectrometer. Quality MS/MS spectra were collected by enabling monoisotopic precursor and charge selection settings, ranging from +2 to +7. Ions with unassigned charge state (0, 1) were excluded. For each MS scan, target ions were detected at a resolution of 70,000 (at m/z 200), maximum injection time for Orbitrap parent scans was 50 ms, allowing 1 microscan. An automatic gain control (AGC) was set as 3e6 with a scan range of 300-1600 m/z. The MS2 parameters were adjusted to a resolving power of 35,000 (at m/z 200), AGC target was set as 1e5 with a maximum injection time of 200 ms, intensity threshold of 2e4 was applied to prevent overfilling of the ion trap, dynamic exclusion of 30 s and 2 Da exclusion width was used. Peptide match

was set as preferred, with the adjustment of fixed first mass set as 100 m/z and the normalized collision energy was set as 32. LC-MS/MS runs and downstream analysis per treatment were performed with four biological and two analytical replicates for liver female and testis, which were analyzed first. In the ovary and liver male, only biological replicates were analyzed, as analytical replicates were not considered necessary, because no differences were observed in liver female and testis.

- **Database search.** The obtained LC-MS/MS raw data were processed using Proteome Discoverer 2.2 software (Thermo Fisher Scientific). For peptide identification, MS data were matched to the zebrafish database obtained from Ensembl (Download version: April. 02. 2017) using the SEQUEST algorithm. A maximum number of 2 missed cleavages per peptide were allowed (Fig. S1A). Mass tolerance for finding peptides was adjusted to 10 ppm and 0.02 Da for matching fragment peaks. Oxidation (M) and Acetyl (N-term) were specified as dynamic modifications, carbamidomethyl (C), TMT-6plex (N-term) and TMT-6plex (K) were specified as static modifications. Strict target false discovery rate (FDR) for highly confident peptide was specified as < 1 % and a relaxed target FDR for peptide hits with moderate confidence was adjusted to < 5 % using a decoy database approach. Co-isolation threshold of 50 and average signal-to-noise ratio above 10 was allowed for reporter ion quantification. Pairwise ratio was considered in ratio calculation and no imputation of missing values was allowed. Normalization was performed to correct experimental bias using total peptide amount (Fig. S1B). Principles of parsimony were enabled in assembling peptides into proteins and protein groups. Proteins were considered as identified if at least two unique peptides were matched. To control errors in proteomic data, downstream statistical comparison of fadrozole-treated samples relative to control was

performed with multiple t-tests (Two-stage Benjamini, Krieger, & Yekutieli test with a FDR of 5%) in GraphPad Prism. A $q < 0.05$ was considered statistically significant.

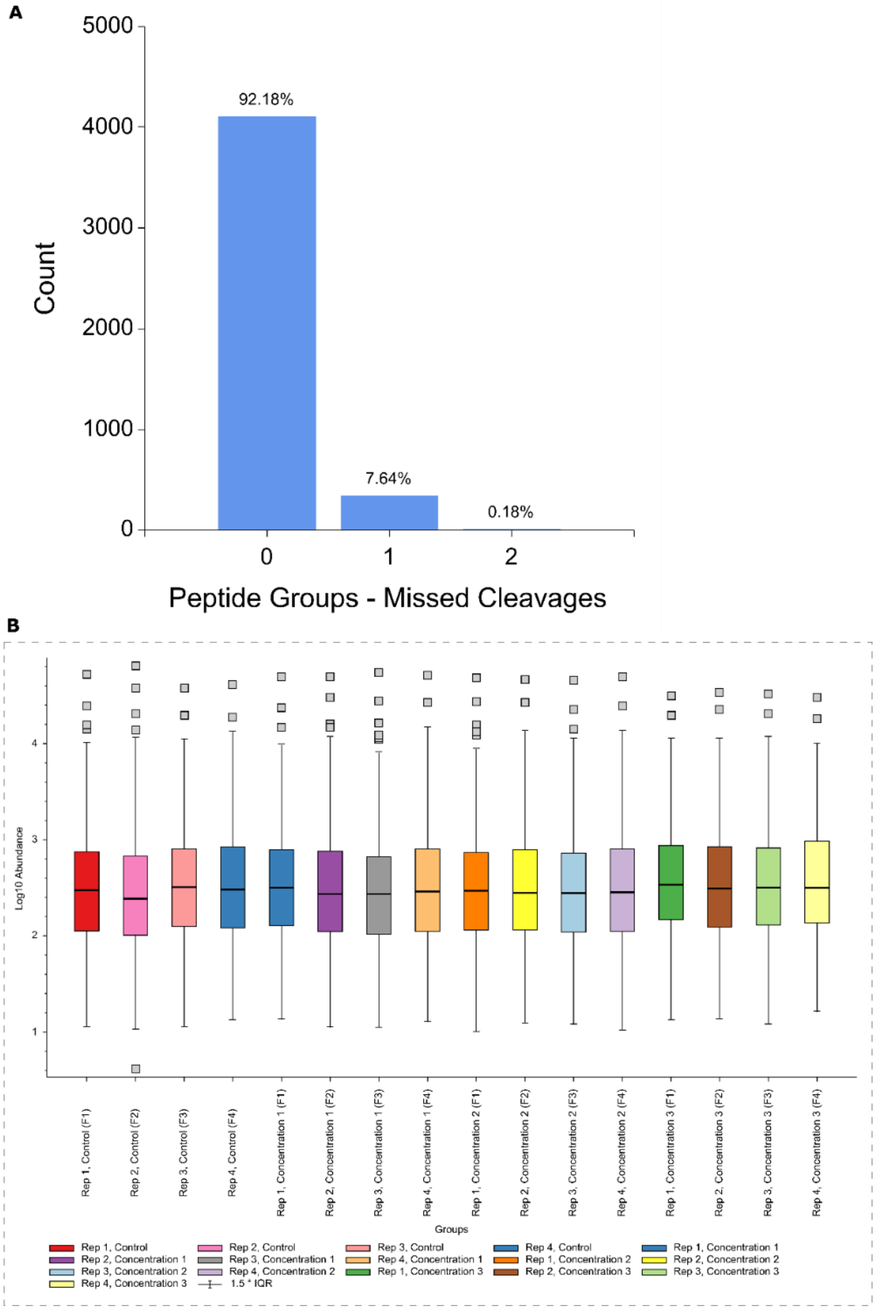

Figure S1: Quality control of mass spectrometry data (A) miss cleavages (B) sample abundance of peptides measured across treatments by TMT labelling

5) **Functional enrichment analysis of biological processes across different tissues.** In order to detect molecular responses and to profile the activities of aromatase inhibition following fadrozole exposure on biological functional processes, an unsupervised enrichment analysis was performed. To this end, lists of differentially identified proteins in the livers and gonads of zebrafish exposed to fadrozole were transferred into FunRich for enrichment analysis. In the female liver, significantly regulated biological processes ($p < 0.05$) following exposure to fadrozole, were 'regulation of estrogen synthesis', 'metabolic process', 'glycolysis', 'tricarboxylic acid cycle (TCA cycle)', 'fatty acid synthesis', 'energy metabolism', and 'response to xenobiotic stimulus' (Fig. S2A). Subsequently, in the female ovary, significantly regulated biological processes were also 'regulation of estrogen signaling', 'metabolic process', and 'fatty acid synthesis'. Additionally, 'microtubule-based process' and 'egg coat formation' were found to be significantly regulated ($p < 0.05$) (Fig. S2B). In the male liver, the strongest regulated biological processes include 'metabolic processes', 'glycolysis', 'estrogen synthesis', 'fatty acid metabolism', 'TCA cycle', 'ATP synthesis', and 'pentose phosphate pathway' ($p < 0.05$) (Fig. S2C), which is in parts similar to the responses found in female liver (Fig. S2A). Biological processes such as 'glycolysis', 'fatty acid beta oxidation', 'microtubule-based process', 'cellular response to estrogen stimulus', 'energy metabolism', as well as 'metabolic process' were strongly affected in the male testis ($p < 0.05$) (Fig. S2D). Under the broad functional categories, 'regulation of cellular response to estrogen stimulus' was common across the different tissues. Thus, the results indicate that exposure to fadrozole affects estrogen synthesis at the molecular level and confirms the observed reduction in plasma VTG levels and decreased egg numbers (Fig. 1 A, D) as well. According to the here described findings, the deficiency in estrogen

signaling has been reported to trigger an increase in fatty acid oxidation [2] and the deregulation of glycolytic proteins and glucose uptake, resulting in decreased aerobic glycolysis [3]. Similarly, a concentration-dependent inhibition of ATP synthesis by exposure to 17 ß-estradiol [4] also indicates the role of estrogen signaling in energy metabolism. Enrichment analysis further indicates that the exposure to fadrozole affects proteins involved in metabolic processes including glycolysis and fatty acid synthesis (Fig. S2). The deficiency in estrogen synthesis has been reported to promote metabolic dysfunction [5,6]. In addition, estrogen synthesis has been documented to induced glucose metabolism through the regulation of pentose phosphate pathway [7], as observed in the enrichment analysis of the male liver and testis (Fig. S2 C, D). An inhibitory effect of estrogen synthesis in microtubule stability has been reported previously [8]. In our study, 'microtubule-based process' was enriched in the female ovary and the male testis following the impairment in estrogen synthesis by fadrozole treatment. In summary, the results of the functional enrichment analysis for significantly regulated biological processes indicates similar sex-specific patterns [9] in enriched biological pathways that suggests an impairment in estrogen synthesis. This is thus in line with the anticipated MoA of aromatase inhibition known for fadrozole (Fig. S3 A-D).

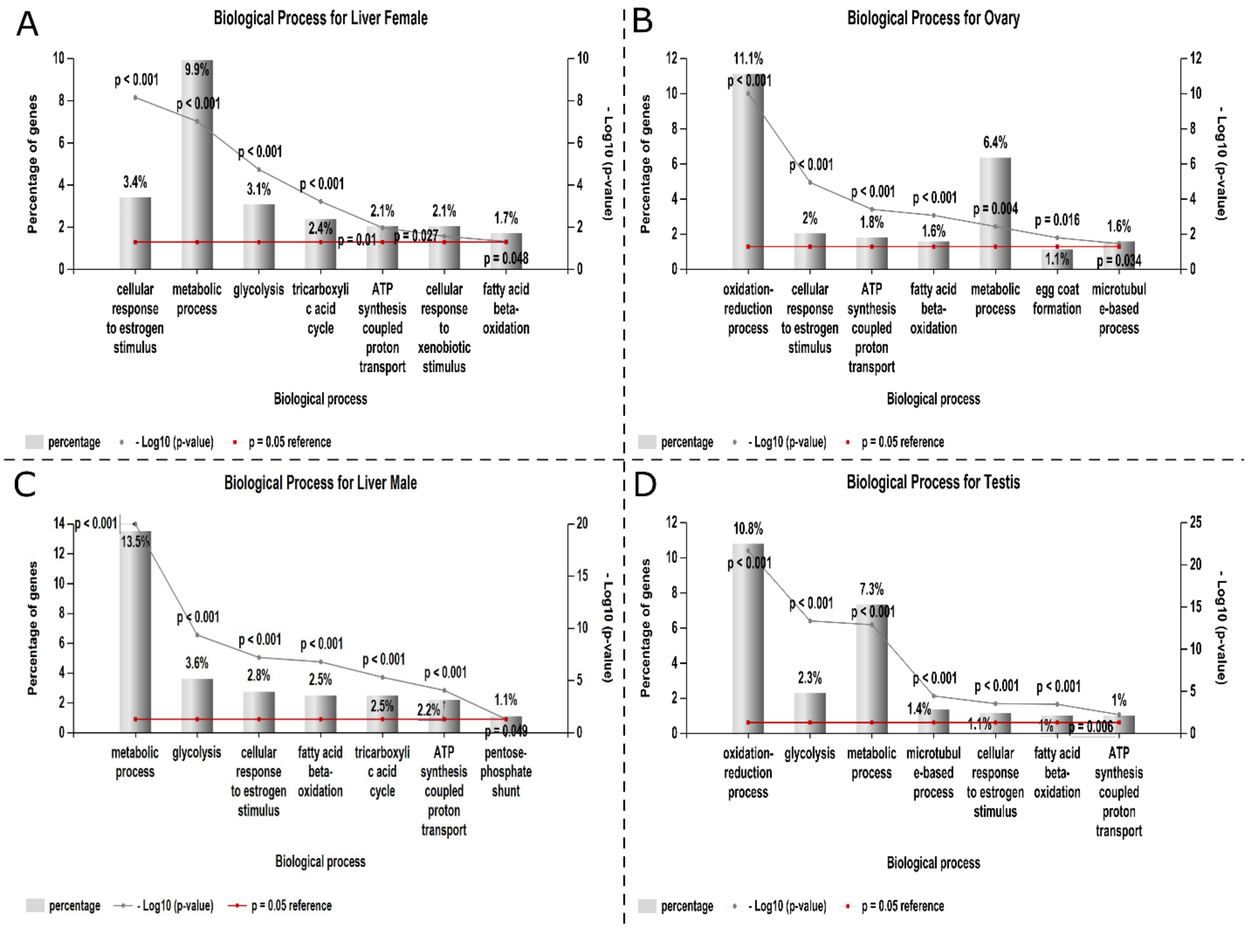

***Figure S2**: Enrichment analysis of regulated functional biological processes following 21days of fadrozole exposure. (A-D) Significantly enriched biological processes in the female liver (A), in the female ovary (B), in the male liver (C), and in the male testis (D). Statistical significant of regulated biological processes were determined with $p \leq 0.05$ in FunRich tools* [10].

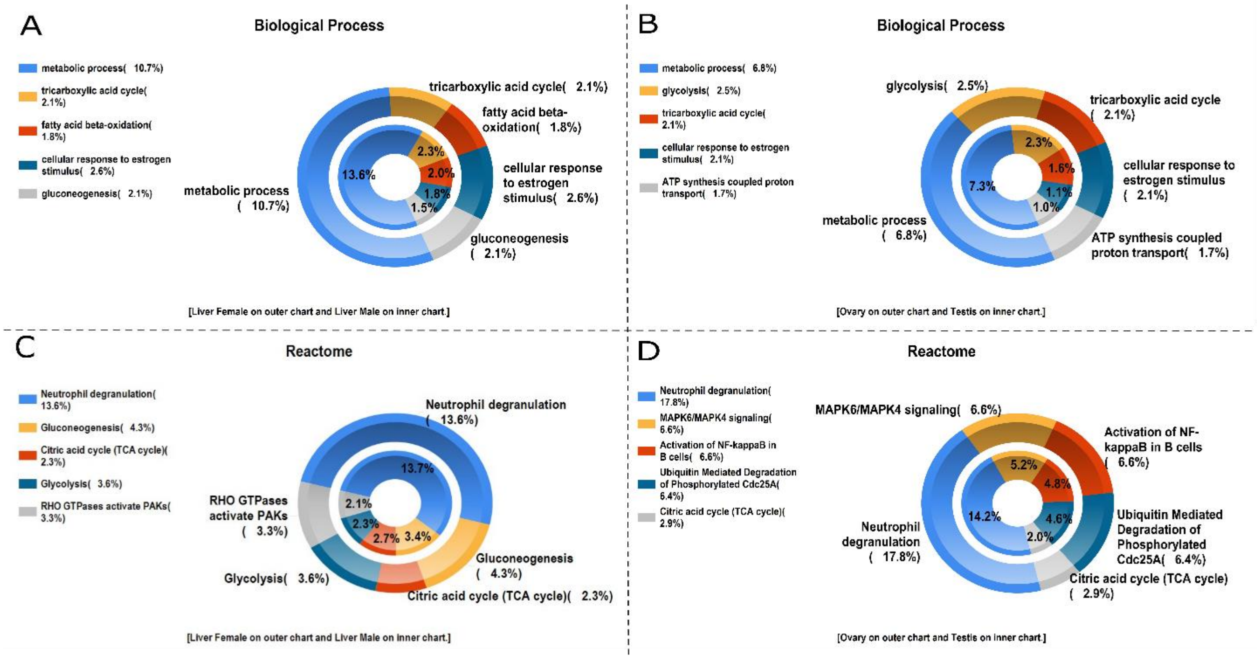

Figure S3: Comparative sex-specific enrichment analysis of regulated functional biological processes and activated pathways following 21 days exposure of zebrafish to fadrozole. (A-B) Percentage distribution of enriched biological processes in the female and male liver (A), and in the female ovary and male testis (B). (C-D) Distribution of protein abundances enriched in biological pathways in the female and male liver (C), and in the female ovary and male testis (D). Statistical significance of regulated biological processes were determined with $p \leq 0.05$ in FunRich tools [10].

Associated Content

Additional supplementary information:

- Proteins detected in proteomics analysis across all replicates for liver female samples (Liver Female.xlsx)
- Proteins detected in proteomics analysis across all replicates for liver male samples (Liver Male.xlsx)
- Proteins detected in proteomics analysis across all replicates for ovary samples (Ovary.xlsx)
- Proteins detected in proteomics analysis across all replicates for testis samples (Testis.xlsx)
- Chemical analysis of fadrozole concentrations (Fadrozole_analysis.xlsx)
- Cumulative number of eggs subsequent to fadrozole treatment (Table S1.xlsx)

References

1 OECD. *Test No. 229: Fish Short Term Reproduction Assay*. (2009).

2 Jensen, M. D., Martin, M. L., Cryer, P. E. & Roust, L. R. Effects of estrogen on free fatty acid metabolism in humans. *American Journal of Physiology-Endocrinology and Metabolism* **266**, E914-E920, doi:10.1152/ajpendo.1994.266.6.E914 (1994).
3 Cai, Q., Lin, T., Kamarajugadda, S. & Lu, J. Regulation of Glycolysis and the Warburg Effect by Estrogen-related Receptors. *Oncogene* **32**, 2079-2086, doi:10.1038/onc.2012.221 (2013).
4 Moreno, A. J. M., Moreira, P. I., Custódio, J. B. A. & Santos, M. S. Mechanism of inhibition of mitochondrial ATP synthase by 17β−Estradiol. *Journal of Bioenergetics and Biomembranes* **45**, 261-270, doi:10.1007/s10863-012-9497-1 (2013).
5 Monteiro, R. *et al.* Estrogen Signaling in Metabolic Inflammation. *Mediators of Inflammation* **2014**, 20, doi:10.1155/2014/615917 (2014).
6 Mauvais-Jarvis, F., Clegg, D. J. & Hevener, A. L. The role of estrogens in control of energy balance and glucose homeostasis. *Endocrine reviews* **34**, 309-338, doi:10.1210/er.2012-1055 (2013).
7 Sun, Y. *et al.* Estradiol promotes pentose phosphate pathway addiction and cell survival via reactivation of Akt in mTORC1 hyperactive cells. *Cell Death & Disease* **5**, e1231, doi:10.1038/cddis.2014.204 (2014).
8 Kipp, J. L. & Ramirez, V. D. Estradiol and testosterone have opposite effects on microtubule polymerization. *Neuroendocrinology* **77**, 258-272, doi:10.1159/000070281 (2003).
9 Liang, X., Feswick, A., Simmons, D. & Martyniuk, C. J. Environmental toxicology and omics: A question of sex. *Journal of proteomics* **172**, 152-164, doi:10.1016/j.jprot.2017.09.010 (2018).
10 Pathan, M. *et al.* FunRich: An open access standalone functional enrichment and interaction network analysis tool. *Proteomics* **15**, 2597-2601, doi:10.1002/pmic.201400515 (2015).
